# Primary Systemic Amyloidosis and High Levels of Angiotensin-Converting Enzyme: Two Case Reports

**DOI:** 10.1155/2013/976379

**Published:** 2013-03-10

**Authors:** J. Praena-Segovia, A. Sanchez-Gastaldo, M. Bernabeu-Wittel, R. Ocete-Pérez, R. Ávila-Polo, M. L. Martino

**Affiliations:** ^1^Internal Medicine Department, University Hospital Virgen del Rocío, Avenida Manuel Siurot s/n, 41013 Seville, Spain; ^2^Oncology Department, University Hospital Virgen del Rocío, Avenida Manuel Siurot s/n, 41013 Seville, Spain; ^3^Radiology Department, University Hospital Virgen del Rocío, Avenida Manuel Siurot s/n, 41013 Seville, Spain; ^4^Pathology Department, University Hospital Virgen del Rocío, Avenida Manuel Siurot s/n, 41013 Seville, Spain; ^5^Haematology Department, University Hospital Virgen del Rocío, Avenida Manuel Siurot s/n, 41013 Seville, Spain

## Abstract

Infiltrative heart diseases are caused by a heterogeneous group of disorders; amyloidosis and sarcoidosis are two frequent causes of myocardial infiltration, which differ in clinical and biological outcome and treatment issues. The presence of high levels of angiotensin-converting enzyme (ACE) in a patient with infiltrative heart disease may increase suspicion of sarcoidosis. Nevertheless, no mention about increased ACE levels in extracerebral primary systemic amyloidosis is available. We present two cases of primary systemic amyloidosis, which are cardiac involvement and elevated ACE levels.

## 1. Introduction

Restrictive cardiomyopathies (RCMs) constitute a heterogeneous group of heart muscle conditions characterized by an increased stiffness of the myocardium that causes a decrease of ventricular filling and cardiac output. The majority of restrictive cardiomyopathies are secondary to a systemic disorder such as amyloidosis, sarcoidosis, scleroderma, haemochromatosis, eosinophilic heart disease, malignancy infiltration, or as a result of radiation treatment [1].

The symptoms of heart failure are dyspnoea and low exercise tolerance. In the physical examination, leg oedemas, ascites, hepatomegaly, and increased central venous pressure can be present. 

The changes in electrocardiogram are often unspecific. Diastolic dysfunction with preserved systolic function is often the only echocardiographic abnormality that may be noted. Restrictive cardiomyopathy is diagnosed based on medical history, physical examination, and tests, such as blood tests, electrocardiogram, chest X-ray, echocardiography, and magnetic resonance imaging. Although there are some typical imaging characteristics of the various conditions that can affect the filling of the heart under the generic classification of restrictive cardiomyopathies, imaging findings are often unspecific and each condition has a spectrum of cardiac manifestations from a very early involvement with mild and subclinical disease to a very severe appearance with huge pathological findings. Echocardiography is a multimodality imaging technique that, when used appropriately and expertly in its full capacity, allows for the comprehensive description of cardiac involvement. Further imaging techniques should be adapted to the perceived clinical suspicion: in the majority of cases, cardiac magnetic resonance imaging will be performed to confirm and describe cardiomyopathies. In rare occasions, the definitive diagnosis of the etiology needs invasive endomyocardial biopsy, due to recent developments in imaging techniques and extracardiac tissues biopsies [2]. 

High levels of plasmatic angiotensin-converting enzyme (ACE) in the presence of an RCM suggests the existence of underlying sarcoidosis [3]. However, increases of plasma ACE do not only occur in this disease, but also in other granulomatous illnesses such as tuberculosis, granulomatous hepatitis, leprosy, silicosis, and asbestosis and even in healthy persons.

We report two patients with RCM, in whom high levels of plasma ACE were present; the final diagnosis in both cases was cardiac amyloidosis as a clinical manifestation of primary systemic amyloidosis associated with the overproduction of immunoglobulin light chains.

## 2. Case 1

A 57 year-old woman was evaluated because of an ischemic chest pain associated to left ventricular hypertrophy with asymmetric thickness and diastolic dysfunction suggestive of infiltrative cardiomyopathy. The patient suffered from hypertension for 27 years since her second pregnancy and from primary hypothyroidism due to Hashimoto's thyroiditis, and she was diagnosed with hypertensive cardiomyopathy two years ago. She was prescribed furosemide, aspirin, atorvastatin, enalapril, atenolol, and levothyroxine. Physical exam was normal except for the presence of rales in both lung basal segments and bilateral malleolar edema.

 All complementary studies were normal including hemogram, coagulation, iron metabolism, and renal, hepatic, and thyroid functions. Tumor markers, serology for antineutrophil cytoplasmic antibody (ANCA), antinuclear antibody (ANA), rheumatoid factor (RF), *β*2 microglobulin, and erythrocyte sedimentation rate (ESR) were normal too. Levels of B-natriuretic peptide and of ACE were elevated, the second one with a value of 112 IU/L. Serum proteinogram and protein immunoglobulins were normal, but monoclonal IgG*λ* band was detected by immunofixation electrophoresis. Levels of kappa-lambda light chains in urine were elevated (*λ*: 124 mg/L, *κ*: 137.5 mg/L). Proteinuria of 2.500 mg per day was found.

Electrocardiogram registered decreased QRS complex voltage. Chest X-ray showed cardiomegaly and mild bilateral pleural effusions. Echocardiography demonstrated severe and asymmetric left ventricular hypertrophy, which was more evident in apical, posterior, and anterior segments. Ejection fraction was normal as well as myocardial velocity gradients, valves, and contractility function.

**Figure 1:**
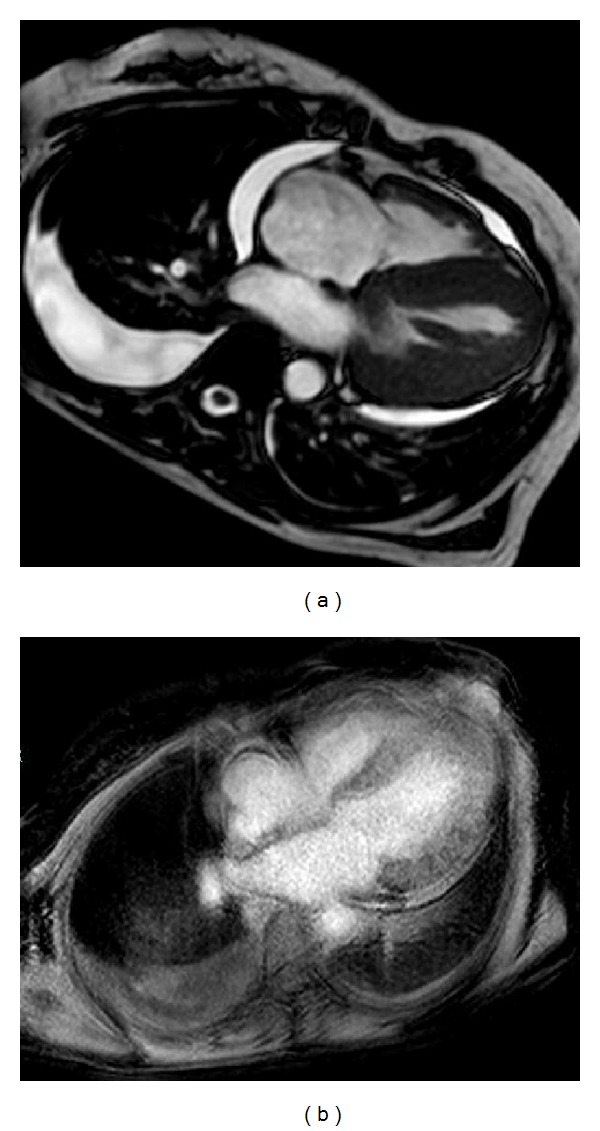
(a) Cardiac MRI shows diffuse infiltration with endomyocardial fibrosis including interatrial septum. Pericardial and pleural effusions demonstrate heart failure. (b) Diffuse and global pattern of late gadolinium enhancement that matches the distribution of amyloid on histology (also exits enhancement at interauricular septum).

A cardiac magnetic resonance study was performed showing left ventricular hypertrophy and thickening of the interatrial septum and right atrial free wall. After gadolinium administration was observed, a diffuse, delayed, and faint enhancement involving all four chambers predominantly in subendocardial areas, findings which are clearly correlated with an infiltrative process and very suggestive of cardiac amyloidosis (Figure 1). An abdominal sonography showed bilateral kidney enlargement with an adequate corticomedullar differentiation.

The etiologic diagnosis was achieved by kidney biopsy. Histopathological study revealed amyloid deposits by Congo red staining and viewing under polarized light a distinctive apple-green birefringence. Tamm-Horsfall proteins were present in tubules. Lambda was high positive by immunochemistry but AA amyloid and Kappa were negative in the study.

The final diagnosis was primary systemic amyloidosis (AL) with cardiac and renal involvement.

At first treatment, she was included in autologous stem cell transplantation (ASCT) program, but it was not possible due to nonmobilization for hematopoietic stem cell (HSC) after plerixafor, so she received treatment with melphalan associated to corticosteroids therapy. Due to complications during the evolution, she suffered an ischemic stroke in cerebral medium artery without neurological deficits after resolution. Inspite of treatment, she developed a nephrotic syndrome with normal kidney function, and because of a progressive increment of proteinuria, melphalan was substituted by bortezomib after the seventh chemotherapy cycle. About the cardiac involvement, a systolic and a severe diastolic progressive dysfunction was shown by echocardiography one year later. 

## 3. Case 2

A 64 year-old-man was evaluated because of a heart stroke. Restrictive cardiomyopathy and interstitial pneumopathy were discovered as incidental findings in the study. He had been asymptomatic before the stroke. The patient suffered from hyperlipidemia. He smoked twenty cigarettes a day and drank alcohol occasionally. His medications included omeprazol and atorvastatin. He had no known allergies to medications. The physical examination only revealed few rales in lungs basal segments through pulmonary auscultation. 

In the complementary studies, we found a light hypoproteinemia, a known hypercholesterolemia, and high levels of ACE (83 UI/L, normal until 55 UI/L). The rest of serum studies were normal including hemogram, renal, hepatic, and thyroid functions, protein immunoglobulins, serum light chains kappa-lambda, beta2 microglobulin, antinuclear antibodies, and erythrocyte sedimentation rate (ESR). Prolonged incubation blood cultures were made just as HIV, HBV, HCV, Brucella and syphilis serology. 

There were elevated levels of immunoglobulin light chains in urine, especially lambda light chain (2.8 mg/dL, being normal until 0.39 mg/dL). Chest X-ray showed a bilateral interstitial infiltrate with Kerley-B lines and some grade of precapillary pulmonary hypertension. This interstitial pattern was confirmed on a thorax high resolution computed tomography that suggested type III sarcoidosis or systemic amyloidosis like most plausible diagnoses. Echocardiography revealed left ventricle hypertrophy with a normal ejection fraction. Cardiac magnetic resonance imaging showed a restrictive pattern with diffuse infiltrative cardiomyopathy expressed by huge wall thickness of both ventricles with a predominant subendocardial distribution. 

**Figure 2:**
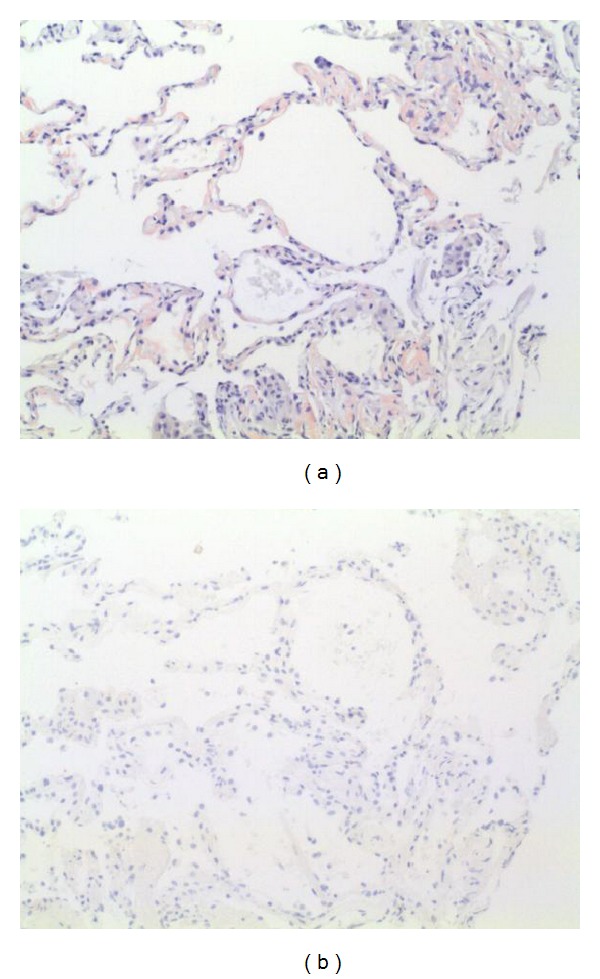
(a) Pulmonary parenchyma with amyloid deposition showed by Congo red staining. 10x. (b) The same image but negative for amyloid immunochemistry. 10x.

As a systemic infiltrative disease was suspected, an abdominal subcutaneous flat biopsy was taken, being normal. Then, we decided to take a bronchial biopsy through a bronchoscopy with bronchoalveolar lavage, which was stained and cultivated to exclude *Pneumocystis jirovecii* and mycobacterial infections. The pathologic study identified extracellular amyloid deposits by Congo red staining and viewing under polarized light where amyloid deposits produce a distinctive apple-green birefringence (Figure 2). The determination of para-aminosalicylic acid (PAS) and AA amyloid in the specimen was negative. So, the diagnosis was systemic AL amyloidosis. 

An ASCT was carried out, previous conditioning with melphalan (140 mg/m^2^ in two doses one day), that was not effective enough (persistent elevated levels of immunoglobulin light chains in urine). The patient suffered a congestive heart failure (CHF) and a light mucositis as side effects, which were solved with pharmacologic treatment. 

Then, it was followed by treatment with bortezomib and prednisone, without melphalan. The patient has received six cycles of this treatment, obtaining complete response with disappearance of immunoglobulin light chains in urine. During these cycles, the patient has suffered multiple episodes of CHF, right predominantly, which have been treated with furosemide. Actually, the possibility of a heart transplantation is being evaluated, due to infiltrative cardiomyopathy with symptomatic CHF.

## 4. Discussion

Our two patients presented a suspected systemic infiltrative disease affecting the heart with signs, symptoms, and results of tests compatible with both amyloidosis and sarcoidosis. In the first case, the patient suffered from ischemic chest pain with a hypertrophic left ventricule in echocardiography, and we discovered a nephrotic syndrome in the tests done. In the second case, the patient was asymptomatic but had a pattern of an interstitial lung disease in chest X-ray and hypertrophic left ventricule in echocardiography as incidental findings. Both of them had elevated levels of serum ACE, which were compatible with sarcoidosis, but in both elevated levels of immunoglobulin light chains in urine were found, which conducted to the AL amyloidosis suspicion. The final diagnosis of both cases required extracardiac tissue biopsies (renal biopsy in case 1 and bronchial biopsy in case 2), which resulted in the definitive diagnosis of AL amyloidosis. 

RCM has not uniformly accepted diagnostic criteria. Unlike other cardiomyopathies, RCM is a functional classification. The classic anatomical features of RCM are small left ventricule with marked atrial dilatation and normal systolic function. The pathophysiological characteristics are those of increased ventricular filling and pressures with a dip-and-plateau pattern in early diastolic pressures traces [2]. RCMs are diagnosed based on medical history, physical examination, blood test, electrocardiogram, chest X-ray, echocardiography, and cardiac magnetic resonance imaging. These last two complementary studies and extracardiac tissues biopsies are usually enough to diagnose the etiology of RCM, so invasive endomyocardial biopsy is performed rarely. 

Amyloidosis refers to a variety of diseases in which an abnormal, insoluble, extracellular protein material (named amyloid) is deposited in organs and tissues. This leads to dysfunction of the organ where it is located. There are different forms of amyloidosis. The primary systemic amyloidosis (AL) is produced by the deposit of immunoglobulin light chains (kappa-lambda) derived from a monoclonal disorder of immune cell function. It may affect all kinds of tissues, except the central nervous system. The most frequently affected organ is the kidney, presenting in most patients as a nephrotic syndrome (as in our case number 1). The second damaged organ in frequency is the heart, and this can occur in 90% of the patients with AL amyloidosis. It is expressed as an RCM, being by far, the commonest cause of RCM [4]. The secondary reactive systemic amyloidosis (AA amyloidosis) is produced by the deposit of an acute phase protein made in the liver in chronic inflammatory diseases. In this form of amyloidosis, the heart involvement is rare, causing less structural and functional impairment than AL amyloidosis. Amyloid-*β* peptide (A*β*) is found in Alzheimer's disease brain lesions causing a cerebral amyloid angiopathy. It is a localized amyloidosis form (A*β* amyloidosis), so it does not affect the heart.

Sarcoidosis is a multisystemic granulomatous inflammatory disease of unknown etiology. The sarcoid granulomas most often appear in the lungs and lymph nodes, but virtually any organ can be affected. Frequent initial symptoms are cough, fatigue, night sweats, and weight loss, but approximately half of the patients are asymptomatic. Symptomatic cardiac involvement is present in 5% of patients, but heart granulomas are found in approximately 25% of autopsy studies [3]. Sarcoidal granulomas produce ACE, which is elevated in 60% of patients with sarcoidosis [2]. However, the value of serum ACE levels in diagnosing or managing sarcoidosis remains controversial. Normal levels have been found in patients with recently diagnosed sarcoidosis, and elevated levels of ACE may be present in other granulomatous diseases such as tuberculosis, silicosis, leprosy, asbestosis, and granulomatous hepatitis. Nevertheless, we have not found any description of elevated ACE levels in AL amyloidosis. At least 5% of the elevated levels of ACE do not have an underlying sarcoidosis, so another diagnosis must be considered [1]. The levels of serum ACE have been used to monitor progress sarcoidosis in response to therapy [5].

With respect to the issue of the concurrence of amyloidosis and increased ACE levels, scare evidence has been stated. It seems that ACE-1 is one of the enzymes degrading the amyloid-*β* peptide in the central nervous system, just as endothelin-converting enzymes (ECE 1 and 2), neprilysin, matrix metalloproteinases, and insulin-degrading enzyme (IDE) [6]. Activity of ACE-1 is reportedly increased in Alzheimer's disease in direct relationship to parenchymal A*β* amyloid load, raising the possibility that intracerebral ACE levels are upregulated as a response to A*β* accumulation within the brain [7]. But we have not found any relationship with AL amyloidosis and serum ACE levels, and we do not know if this fact would have the same pathophysiological significance as in A*β* amyloidosis. So, the real pathophysiological relationship between high serum ACE levels and AL amyloidosis is unknown. It would be very interesting to assess how often this finding is present in patients with primary amyloidosis in order to establish its pathophysiological ways and importance in the diagnosis and course of the disease.

After these findings and the possible pathophysiological explanation, we suggest clinicians to include primary systemic amyloidosis in the differential diagnosis of a suspected infiltrative multiorganic disease, also when increased serum ACE levels are present.

## Abbreviations

ACE: Angiotensin-converting enzyme
RCM: Restrictive cardiomyopathy
AL: Primary systemic amyloidosis
ASCT: Autologous stem cell transplantation
CHF: Congestive heart failure
PAS: Para-aminosalicylic acid.
